# Improvement of Hot Tearing Resistance of AZ91 Alloy with the Addition of Trace Ca

**DOI:** 10.3390/ma16103886

**Published:** 2023-05-22

**Authors:** Hongchen Xiang, Wenjun Liu, Qiang Wang, Bin Jiang, Jiangfeng Song, Hang Wu, Nan Feng, Linjiang Chai

**Affiliations:** 1 College of Materials Science and Engineering, Chongqing University of Technology, No. 69, Hongguang Road, Banan District, Chongqing 400054, China; xianghongchen@stu.cqut.edu.cn (H.X.); 15086618645@163.com (Q.W.); chailinjiang@cqut.edu.cn (L.C.); 2 College of Materials Science and Engineering, Chongqing University, No. 174, Shazheng Street, Shapingba District, Chongqing 400044, China; jiangfeng.song@cqu.edu.cn; 3 Chongqing Tsingshan Industrial, No. 88, Qingshan North Road, Bishan District, Chongqing 402761, China; wuhang@tsingshan.com (H.W.); fengnan@tsingshan.com (N.F.)

**Keywords:** AZ91, hot tearing susceptibility, solid-solution strengthening, Al_2_Ca, kernel average misorientation

## Abstract

Hot tearing is the most common and serious casting defect that restricts the light weight and integration of magnesium alloy components. In the present study, trace Ca (0–1.0 wt.%) was added to improve the resistance of AZ91 alloy to hot tearing. The hot tearing susceptivity (HTS) of alloys was experimentally measured by a constraint rod casting method. The results indicate that the HTS presents a ν-shaped tendency with the increase in Ca content, and reaches its minimum value in AZ91–0.1Ca alloy. Ca is well dissolved into α-Mg matrix and Mg_17_Al_12_ phase at an addition not exceeding 0.1 wt.%. The solid-solution behavior of Ca increases eutectic content and its corresponding liquid film thickness, improves the strength of dendrites at high temperature, and thereby promotes the hot tearing resistance of the alloy. Al_2_Ca phases appear and aggregate at dendrite boundaries with further increases in Ca above 0.1 wt.%. The coarsened Al_2_Ca phase hinders the feeding channel and causes stress concentration during the solidification shrinkage, thereby deteriorating the hot tearing resistance of the alloy. These findings were further verified by fracture morphology observations and microscopic strain analysis near the fracture surface based on kernel average misorientation (KAM).

## 1. Introduction

Magnesium alloy has received increasing attention due to its low density, high specific strength, excellent shock absorption and electromagnetic shielding properties [1,2,3]. It has important applications in lightweight component products such as shells and brackets, in the form of castings that can simultaneously satisfy the requirements of integrated design and low mass-production costs [4,5]. It is worth noting that both castings and deformed parts prepared on the basis of casting require good casting quality support. As a fatal defect in the casting process, hot tearing resistance has become an important indicator for evaluating casting properties.

In general, hot tearing happens at the final stage of solidification, mainly due to stress concentration and limited feeding ability [6,7,8]. As the solidification process progresses, the solidification shrinkage stress in the alloy increases. When shrinkage stress exceeds the ultimate bearing capacity of the local dendritic microstructure near solidus temperature, microcracks will form. If there is not enough residual melt to heal it at this time, it will further promote the nucleation and propagation of cracks. Macroscopically, hot tearing mainly occurs in the hot spot area or cross-section mutation area due to significant changes in solidification shrinkage stress. It follows from the above that hot tearing involves internal factors [9,10], such as solidification interval, secondary phase, solidification volume shrinkage, load-bearing capacity of the mushy zone microstructure, and eutectic content and distribution, as well as external factors [11,12,13], like the casting process and casting geometry. The various influencing factors are both independent and interact with each other, promoting a very complex hot tearing behavior in a synergistic manner. This is why the phenomenon of hot tearing is so common and difficult to eliminate in casting components. Based on this, extensive research has been undertaken to understand its characteristics and mechanisms. Alternatively, process optimization [14] such as increasing the cooling rate, pouring temperature and mold temperature; electromagnetic field treatment [15,16]; modification melt treatment [17]; or alloying [18,19,20,21] have been used to improve the hot tearing resistance of alloys. Among these methods, alloying is recognized as the most effective method to date. It can optimize the microstructure and solidification behavior. Previous studies [20,21,22,23] reported that addition of rare earth elements, like Gd and Y, to magnesium alloys can reduce the hot tearing susceptibility (HTS) of the alloys. The formation of high-melting-point compounds and long-period stacking-ordered (LPSO) phase helps to refine the microstructure and decreases the solidification interval, and also improves the high-temperature strength of the alloy. For instance, Zhou and co-workers [20] investigated the effect of LPSO phase on hot tearing in Mg–1Zn–*x*Y (*x* = 1, 2, 3 wt.%) alloy and found that the HTS declined with the increase in LPSO content. The lowest HTS was found in Mg–1Zn–3Y alloy. Wei et al. [23] used Gd to replace Y to reduce the HTS of Mg_96.94_–Zn_1_–Y_(2−x)_–Gd*_x_*–Zr_0.06_ (*x* = 0, 0.5, 1, 1.5, 2 at.%) alloy and obtained the lowest HTS in the alloy with the highest Gd content. Numerous high-plasticity LPSO phases provided by increasing Gd pin and bridge at grain boundaries result in microstructure refinement and difficulty in crack nucleation and propagation. Hence, HTS of magnesium alloys can be reduced by abundant rare earth element addition; however, this leads to an increase in density and production cost, and thereby limits its large-scale application in lightweight components.

Attempts to introduce non-rare earth elements, especially potential grain refiners [21,24,25] like Ca, Sr and Si, to minimize or even eliminate hot tearing defects have been ongoing. Ravindran et al. [25] significantly reduced the HTS of AZ91E alloy by adding 1.5 wt.% Si. Both grain size and solidification interval decreased after Si addition. Unfortunately, the mechanical property of the alloy at room temperature is also reduced owing to the formation of Chinese script Mg_2_Si particles. Research on Ca [26,27] shows that it can simultaneously refine microstructure and improve high-temperature creep resistance of magnesium alloys. HTS of alloys can be effectively controlled by their high-temperature creep resistance [28,29]. Therefore, Ca has become one of the preferred elements for suppressing the HTS of magnesium alloys. Tang et al. [24] studied the AZ91D–*x*Ca (*x* = 0.1, 0.3, 0.5, 0.8, 1.0 wt.%) using a critical diameter method, and found that the HTS actually increased with the increase in Ca content. Hot tearing resistance of the alloy was significantly deteriorated after Ca addition exceeded 0.3 wt.%. The increase in HTS was attributed to the decrease in back-filling ability, which resulted from the increase in divorced eutectic formation tendency and the phase transition temperature of the Al_2_Ca phase. Similar results were found by Hort et al. [30] in AZ91D–*x*Ca (*x* = 0.5, 1.5, 2.5 wt.%) alloys. Different from previous research [24,28,30,31], a ν-shaped tendency with the increase in Ca content in Mg–5Al–*x*Ca (*x* = 0.5, 1.0, 2.0, 3.0, 4.0, 5.0 wt.%) alloys was observed by Wang and co-workers [32], and the lowest HTS appeared at a higher Ca content of Mg–5Al–4Ca alloy. However, it should be noted that in the Mg–Al–Ca alloy [31], the HTS decreases with the increase in Ca/Al ratio. A large Ca/Al ratio brought about a mass of eutectic phase and dendrite-skeleton voids, as well as coarsened grains. How did the differences brought about by Ca additions arise, and which is the most important influencing factor in diverse situations? The effect of Ca addition on hot tearing behavior of magnesium alloys is mainly focused on the solidification interval and eutectic content. Research on eutectic distribution, solid solution and intermetallic compounds brought by alloying, as well as the characteristics and distribution of metal compounds is rare, and very few studies have provided a comprehensive analysis.

AZ91 magnesium alloy, the most commonly used die-casting magnesium alloy, is prone to hot tearing during the casting process due to its large solidification interval, high linear expansion coefficient and severe segregation of intermetallic compounds at the grain boundaries. Furthermore, the problem of hot tearing becomes increasingly serious with the development of thin-walled structures and complex shapes of lightweight components. Therefore, the aim of the present work was to study the effect of Ca on the hot tearing resistance of AZ91 magnesium alloy. The solidification behavior, microstructure, content and distribution of eutectics, and the characteristics and distribution of solid solution and intermetallic compounds were characterized and related to hot tearing. It is of great significance to deeply understand the mechanism of the factors affecting alloy hot tearing for improving the hot tearing resistance of magnesium alloys through alloying.

## 2. Experimental Procedures

### 2.1. Materials

AZ91–*x*Ca (*x* = 0.05, 0.1, 0.5 and 1.0 wt.%) alloys were prepared by permanent mold casting. The raw materials of pure Mg (99.99%), Al (99.99%), Zn (99.99%) and Mg–10Mn, Mg–30Ca (all in wt.%) master alloys were cleaned and weighed to obtain the required alloy component. Firstly, pure Mg was melted in a stainless-steel crucible at 700 °C. When the Mg was completely melted, pure Al, Zn, Mg–10Mn and Mg–30Ca master alloys were immediately added to the melt. Meanwhile, the melt temperature was increased to 720 °C and kept about 30 min to obtain a melt of highly uniform composition for each alloy. The melt was refined at this temperature by using hexachloroethane, followed by stirring and slagging. Subsequently, the melt temperature was further increased to 740 °C and held for 20 min to ensure slag liquid separation, followed by slagging. Finally, the melt was cooled to 720 °C and then poured into a hot tearing mold preheated at 250 °C for solidification. The whole melting and pouring process was protected with a mixed gas of SF_6_ (volume fraction between 0.3 and 0.5%) and CO_2_. In addition, raw materials, refining agents and boron-nitride-coated crucible and mold were preheated to remove water. The chemical composition of each alloy was measured by inductively coupled plasma (ICP) spectroscopy [33,34], and the average of three tests was used as shown in Table 1.

### 2.2. Hot Tearing Tests

**Figure 1:**
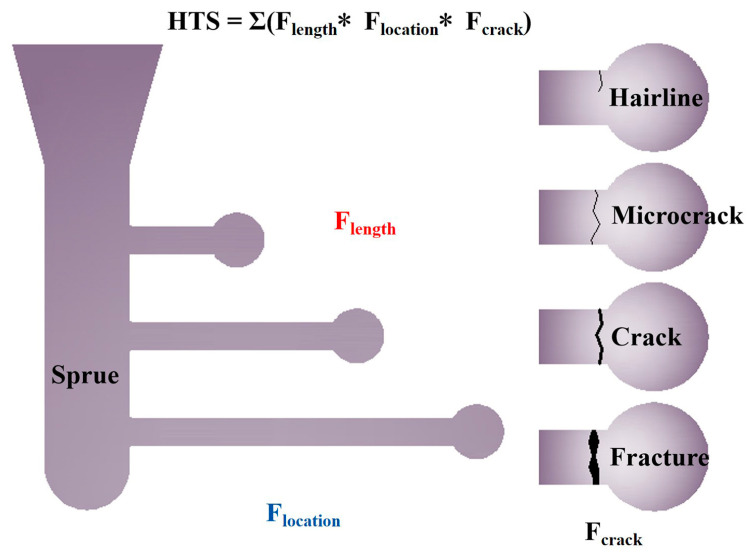
A schematic illustration of hot tearing sample and its assessment method.

The same assessment method of hot tearing susceptivity (HTS) from our previous research [35,36] was used, as shown in Figure 1. Hot tearing samples were prepared in a constraint rod casting (CRC) mold, which had three cylindrical rods of different lengths. Influence factors of rod length, crack location and crack size were introduced to calculate the HTS of alloy, where F_length_ is the length influence factor, with 16 for the shortest rod, 8 for the medium-length rod and 4 for the longest rod; F_location_ is the location influence factor, where 1, 2, 3 represent the location of the cracks observed at the sprue end, the spherical end and the middle position of the cylindrical rod, respectively; and F_crack_ is the crack size influence factor, with 0.5 for hairline, 1 for microcrack, 2 for crack and 3 for fracture. The severity and location of cracks were quantified using a digital camera with close-up lens and bellows, and HTS was the sum of the obtained values. The value of HTS for each alloy was the average of three repeated experiments.

### 2.3. Microstructural Characterization

Small sample blocks were sectioned longitudinally along the centerline of the rods. After grinding and polishing, the samples were etched using a solution of nitric acid alcohol with a volume concentration of 4% for about 10 s. The cracked area near the hot spot and the crack-free area away from the hot spot of samples were examined via optical microscopy (Leica DMI5000M, Wezler, Germany) to obtain the evolution of microstructure. The etched samples near the fracture surface were also used to analyze the eutectics and secondary phase by scanning electron microscopy (JEOL JCM-7000, Tokyo, Japan) equipped with energy dispersive X-ray spectroscopy (OXFORD AZtec X-Max 50, Oxford, UK). In order to characterize the hot tearing behavior, fracture morphology and secondary cracks were examined by scanning electron microscopy, and microscopic strains near the fracture surface were analyzed based on kernel average misorientation. Samples near the fracture surface were polished for 50 min by argon ion polishing (Fischione1061) at 6 kV after grinding. Detailed microstructural features were characterized using a field emission scanning electron microscope (Zeiss Sigma HD, Oberkohen, Germany) equipped with an electron backscatter diffraction (EBSD) system. EBSD analysis was examined by a selected scanning area of 350 × 250 μm^2^ at a step size of 0.8 μm. HKL Channel 5 software (Aztec 3.1) was employed to analyze the inverse pole figure (IPF) and kernel average misorientation (KAM). The KAM is defined as the average misorientation between each measurement point and the nearest neighbors. The third nearest neighbor was used to calculate the KAM at a specific measurement point, and misorientations exceeding a critical value of 5° were excluded in the calculation. In this study, the microscopic strain of microstructure near the fracture surface was evaluated by the average spatial distribution of KAM values. For further phase identification of the alloy, X-ray diffraction (Panalytical Empyrean series 2, Almelo, Netherlands) was employed with a diffraction angle ranging from 20° to 90° at a step size of 0.0131° under Cu–Kα radiation. The solidification process of AZ91 alloy with different Ca addition was calculated by Pandat.

## 3. Results

### 3.1. Hot Tearing Susceptibility (HTS)

**Figure 2:**
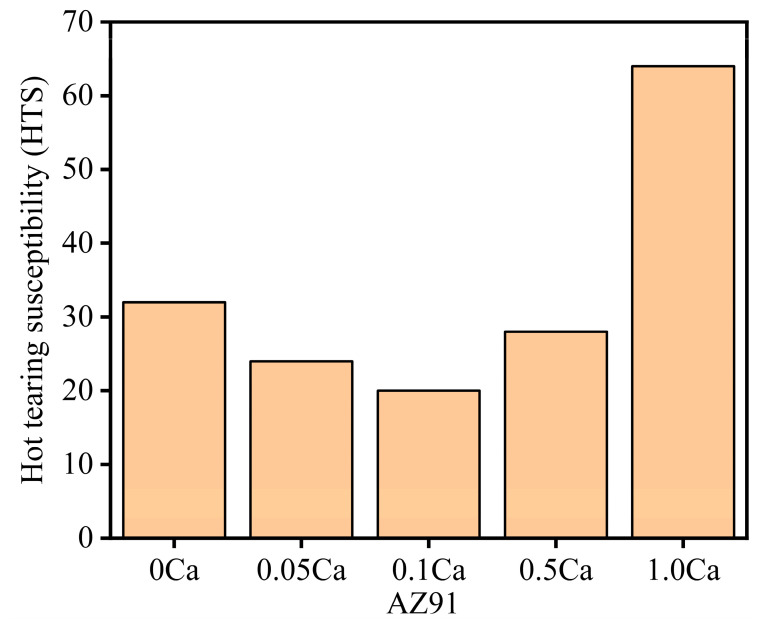
HTS of the AZ91 alloy with different Ca addition.

Figure 2 shows the hot tearing susceptibility (HTS) of AZ91–*x*Ca (*x* = 0.05, 0.1, 0.5 and 1.0 wt.%) magnesium alloy. The HTS presented a ν-shaped tendency with the increase in Ca content. It first decreased and reached the minimum value at 0.1 wt.% Ca. Then, a slight increase in HTS occurred at Ca additions between 0.1 and 0.5 wt.%, followed by a rapid increase when the Ca addition reached 1.0 wt.%. Hot tearing resistance of AZ91 alloy was improved by trace Ca addition. The HTS of AZ91–0.1Ca alloy was 37.5% lower than that of AZ91 alloy. However, when the content of Ca exceeded 0.5%, hot tearing resistance of AZ91 alloy deteriorated significantly. The HTS of AZ91–1.0Ca alloy was 100% higher than that of AZ91 alloy.

### 3.2. Microstructure Evolution

**Figure 3:**
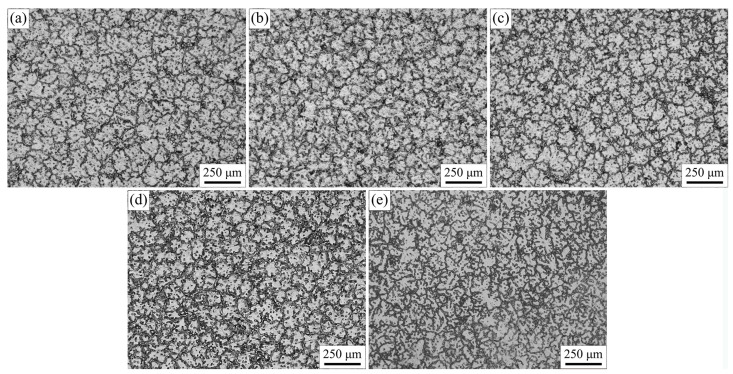
Microstructure away from the hot spot of (**a**) AZ91, (**b**) AZ91–0.05Ca, (**c**) AZ91–0.1Ca, (**d**) AZ91–0.5Ca, (**e**) AZ91–1.0Ca.

**Figure 4:**
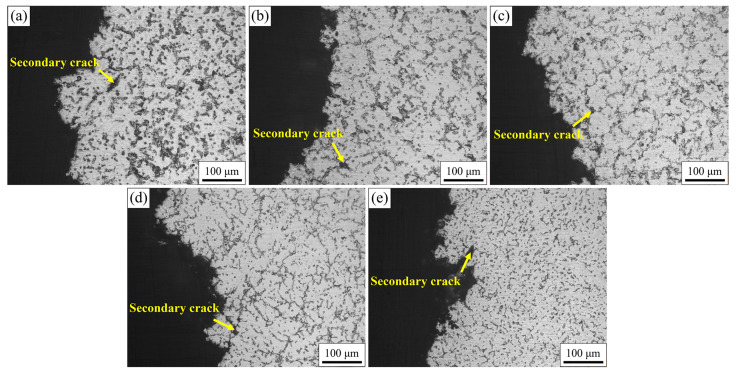
Microstructure near fracture surface of (**a**) AZ91, (**b**) AZ91–0.05Ca, (**c**) AZ91–0.1Ca, (**d**) AZ91–0.5Ca, (**e**) AZ91–1.0Ca.

Hot tearing behavior of an alloy is closely associated with its microstructure characteristics. The microstructure away from the hot spot and near the fracture surface were inspected, as shown separately in Figure 3 and Figure 4. The microstructure of all alloys was composed of gray–white matrix and black secondary phase. With the addition of Ca, the microstructure away from the hot spot was slightly refined with a rose-like morphology, and sphericity at a content below 0.5 wt.%. The coarsening phenomenon with obvious dendrite morphology was observed with further increases in Ca content in AZ91–1.0Ca alloy.

Different from the clear grain boundary presentation of the crack-free region shown in Figure 3, only the dendrite structure was observed near the fracture surface in Figure 4. This was due to the very severe segregation of alloying elements in the final solidified region. Some new secondary phases with needle and granular morphology were introduced by Ca addition. The new phase interrupted the semi-continuous reticulated distribution of Mg_17_Al_12_ [37] phase and formed in its vicinity. With the increase in Ca content, the amount of Mg_17_Al_12_ phase decreased gradually, while the amount of new secondary phase increased. In addition, some secondary cracks and healed cracks close to the fracture surface were observed along the dendrite boundaries.

### 3.3. Fracture Surface Morphology

**Figure 5:**
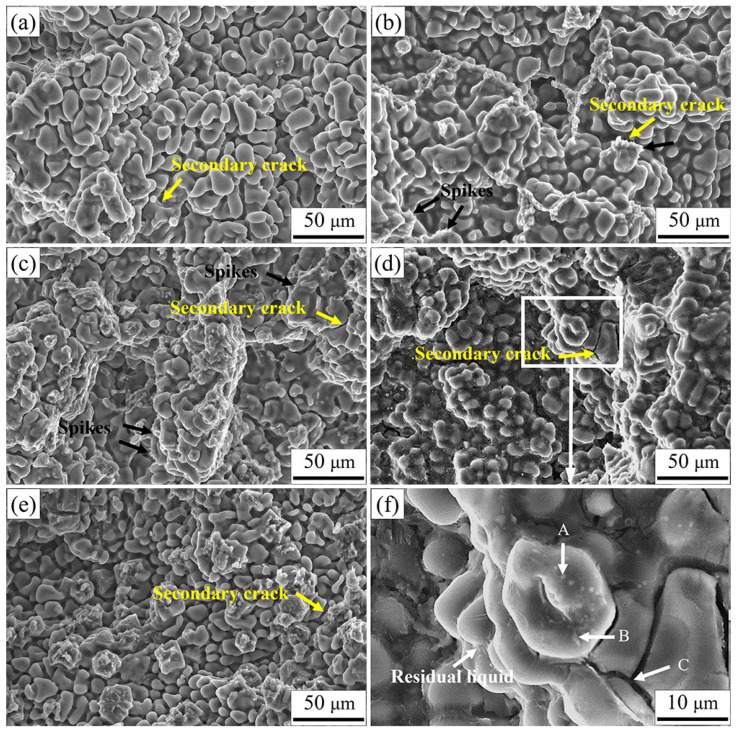
Fracture morphology of the (**a**) AZ91, (**b**) AZ91–0.05Ca, (**c**) AZ91–0.1Ca, (**d**) AZ91–0.5Ca, (**e**) AZ91–1.0Ca; (**f**) local enlargement of (**d**).

Figure 5 shows the fracture morphology of AZ91 alloys with different Ca addition. The “granular” structure was composed of a large number of dendritic branches and intergranular bridging zones (including tearing edges and spikes), as well as secondary phase and secondary cracks (separated liquid film). The dendrite was first refined and then coarsened with the increase in Ca content. This was in accordance with the results of Figure 3. New secondary phase with needle-like and granular morphology was clearly observed in the AZ91–0.5Ca (wt.%) alloy. To understand the feature of secondary phase and micro cracks, EDS analysis was employed in the enlarged area of Figure 5d, and the results are shown in Figure 5f and Table 2. Obviously, Al was enriched in the secondary phase, distributed at dendrites and among interdendritic regions, accompanied by the enrichment of Mn (point A in Figure 5f), Ca and Zn (point B). White particles at dendrites may be the Al_4_Mn phase [38,39], while the white bars may be composed of the Al_2_Ca and Mg_17_Al_12_ phases, based on atomic percentages. Nevertheless, Al deficiency occurred in the crack zone with a large amount of Ca and Zn enrichment (point C). This indicated that there was not enough low melting point Mg_17_Al_12_ phase to feed in time in the final solidification region, and then cracks appeared. Therefore, hot tearing fracture surface consists of free dendritic plane, solute-rich secondary phase, tearing edges, spikes and microcracks.

### 3.4. Observation of Secondary Phase

**Figure 6:**
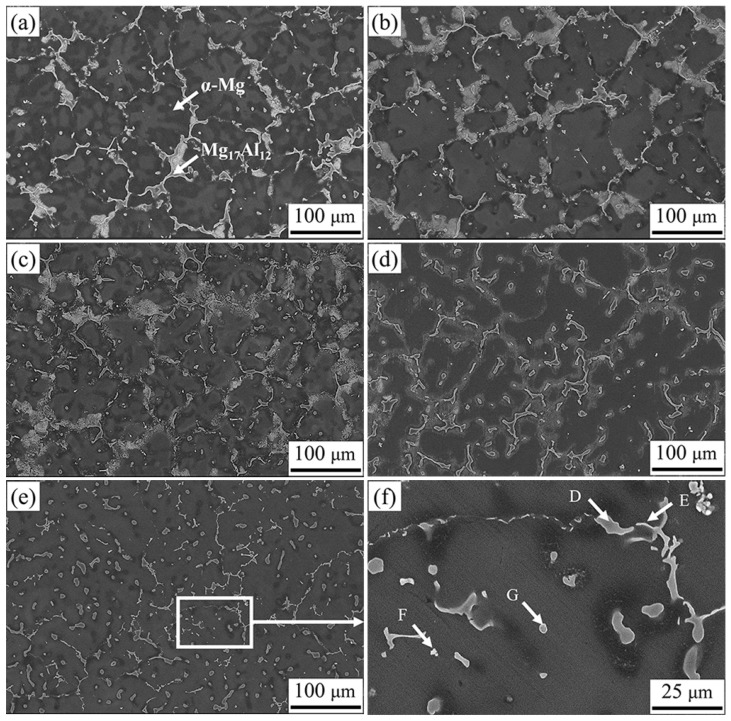
The distribution of secondary phase near the fracture surface of (**a**) AZ91, (**b**) AZ91–0.05Ca, (**c**) AZ91–0.1Ca, (**d**) AZ91–0.5Ca, (**e**) AZ91–1.0Ca; (**f**) local enlargement of (**e**).

**Figure 7:**
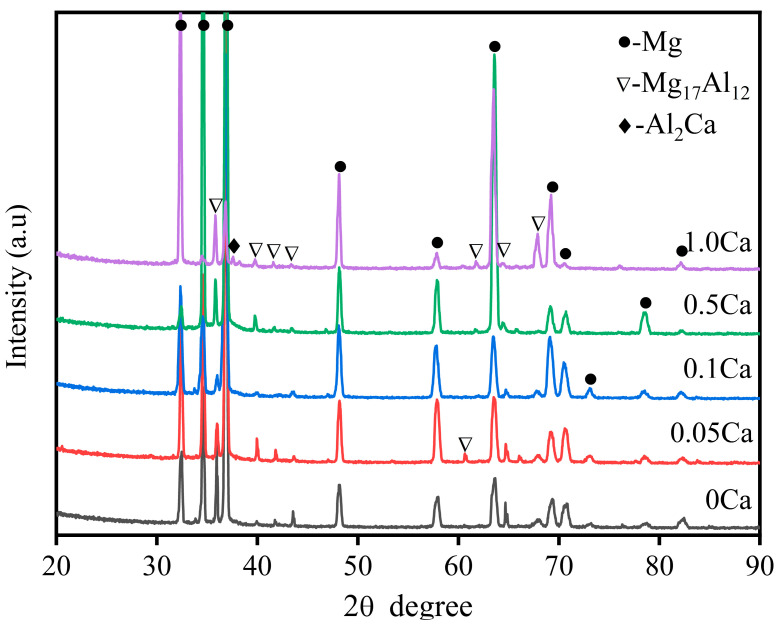
X-ray diffraction analysis of AZ91 alloys with different Ca addition.

To further characterize the composition and distribution of the phases in the structure, a secondary phase analysis was performed for alloys with different Ca content, as shown in Figure 6. The semi-continuous network second phase is visibly refined, while the granular and needle-like second phases appeared nearby in some areas as the Ca content increased. When the Ca content increased to 1.0 wt.%, the continuity of the semi-continuous phase was almost completely destroyed by granular and needle-like secondary phases, in agreement with the phenomenon observed in Figure 4. Magnification and EDS results of local area of AZ91–1.0Ca alloy are shown in Figure 6f and Table 3, respectively. Only α-Mg can be detected in the dark black matrix, and the Mg_17_Al_12_ phase was precipitated in gray–white granular and chain structures. The needle-like phase and white circular particles were thought to be Al_2_Ca [40] and Al_4_Mn, respectively. The XRD results in Figure 7 further confirm the above speculation. The AZ91 alloy is composed of α-Mg and Mg_17_Al_12_ phases. There were no changes in phase composition until the Ca content increased to 0.1 wt.%. The Al_2_Ca diffraction peak first appeared at the addition of 0.5 wt.% Ca, and its intensity increased with the increase in Ca content. Moreover, the diffraction peak of α-Mg and Mg_17_Al_12_ phases shifts slightly to the left. Owing to the large radius of Ca atoms, it dissolves into the α-Mg matrix and the Mg_17_Al_12_ phase increases its lattice constant, resulting in a decrease in the diffraction angle [41].

## 4. Discussion

### 4.1. Solidification Behavior

**Figure 8:**
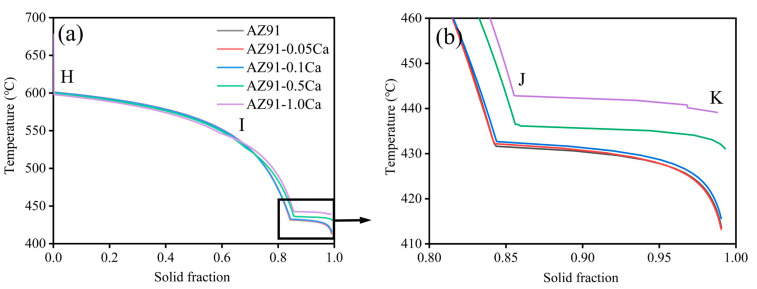
(**a**) Solidification curves of AZ91 alloys with Ca addition; (**b**) local enlargement of (**a**).

The solidification process of AZ91 alloys with different Ca addition was analyzed by Pandat software (https://computherm.com/) as shown in Figure 8. The solidification of alloys undergoes several phase transitions with the decrease in temperature, evident in the different turning points of curves. Combined with the XRD results in Figure 7, the segments of H to I, I to J and J to K separately represent the phase transition of L→α-Mg, L→α-Mg+Al_2_Ca and L→α-Mg+Al_2_Ca+Mg_17_Al_12_. The data of each turning point and calculated solidification interval are presented in Table 4. As the Ca content increases, the liquidus moves down slightly and the solidus moves up, which results in the decrease in the solidification interval of alloys. The variation in the solidification interval is negligible until the Ca content increases to 0.5 wt.%. Noticeable changes appear both in AZ91–0.5Ca and AZ91–1.0Ca alloys, with a clear Al_2_Ca phase transition (point I) on the solidification curve. The solidification interval of AZ91–1.0Ca with the highest HTS (from Figure 2) is the smallest, which is counter-intuitive. Thus, there must be more important factors than the solidification interval affecting the hot tearing behavior of alloys. Furthermore, the mole fraction of solids at eutectic transformation (point J) apparently increased, which means that the eutectic content decreases at the end of solidification in both AZ91–0.5Ca and AZ91–1.0Ca alloys. The decrease in eutectic content will increase the HTS of alloys with restricted feeding ability for cracks during solidification [41,42]. The prediction is consistent with the experimental results of HTS. The thermodynamic calculation of eutectic content shows better prediction ability in the evaluation of HTS of alloys.

### 4.2. Hot Tearing Behavior

It is well known that the Ca add to AZ91 alloy will exist in the form of solid solution and high-melting-point Al_2_Ca phase with different additions [27,31,40]. When the content of Ca does not exceed 0.1 wt.%, it is well dissolved into α-Mg matrix and Mg_17_Al_12_ phase. This is supported by the shift to the left of the diffraction peaks of AZ91 alloys after Ca addition in Figure 7. The solid-solution behavior of Ca could strengthen both the dendrites and the vulnerable area between dendrites without affecting the solid-solution strengthening of Al. The microstructure of alloys is thus refined, which is consistent with the microstructural analysis in both Figure 3 and Figure 5. The high-melting-point Al_2_Ca phases appear at a Ca content of 0.5 wt.% and increase in quantity with further increases in Ca to 1.0 wt.%, shown in Figure 7. As a potential grain refiner, Al_2_Ca has an atomic mismatch of less than 10% with the α-Mg matrix, which can provide heterogeneous nucleation particles for the matrix [26]. The grain size of Ca-containing alloys is smaller than that of AZ91 alloy with an approximate reduction of 42.6% (0.05 wt.% Ca), 38.6% (0.1 wt.% Ca), 33.5% (0.5 wt.% Ca), and 1.7% (1.0 wt.% Ca), respectively, as shown in Figure 8a. With the increase in Al_2_Ca phase caused by excessive consumption of Al, the refinement degree of Ca on alloy microstructure decreases significantly. Previous studies [12,43,44,45,46] have demonstrated that grain refinement can improve the strength of dendrite microstructure and increase the feeding efficiency of eutectic liquid phase through a shorter path between dendrites, thereby reducing the HTS of alloys. However, the AZ91–0.05Ca alloy with the smallest grain size did not have the best hot tearing resistance, while the AZ91 with the largest grain size did not have the worst hot tearing resistance in this study. Therefore, there are more important factors than grain size that determine the HTS of alloys.

**Figure 9:**
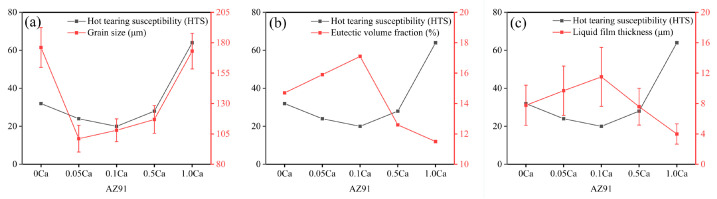
The relationship between HTS and (**a**) grain size, (**b**) eutectic volume fraction and (**c**) liquid film thickness of AZ91 with Ca addition.

Variation in eutectic volume fraction and liquid film thickness from Figure 6 was plotted to study the factors influencing hot tearing behavior of alloys, as shown in Figure 9b,c. Both the eutectic volume fraction and liquid film thickness present a ∧-shaped tendency with the increase in Ca content. With the addition of Ca, eutectic content increases and reaches its maximum value at a Ca content of 0.1 wt.%, at which point the alloy has the lowest HTS. The eutectic volume fractions of AZ91–0.05Ca and AZ91–0.1Ca alloys increase by 8.2% and 16.3% compared to AZ91, respectively. When the Ca content exceeds 0.1 wt.%, eutectic content rapidly decreases and reaches its minimum value at a Ca content of 1.0 wt.%, at which point the alloy has the highest HTS. The eutectic volume fractions of AZ91–0. 5Ca and AZ91–1.0Ca alloys decrease by 14.3% and 21.8% compared to AZ91, respectively. Moreover, the liquid film thickness maintains the best corresponding change relationship with the HTS of alloys. Compared with the liquid film of AZ91 alloy of 7.8 μm, AZ91-0.05Ca and AZ91–0.1Ca show relatively thick liquid films of 9.7 μm and 11.5 μm, while AZ91-0.5Ca and AZ91–1.0Ca display relatively thin liquid films of 7.6 μm and 4.0 μm, respectively.

**Figure 10:**
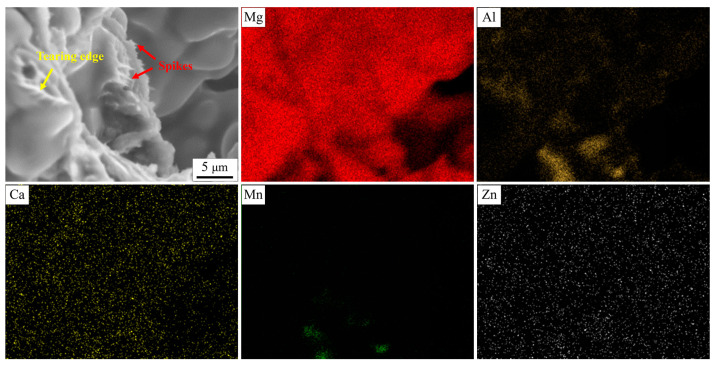
Energy spectrum analysis of the fracture surface of AZ91–0.1Ca alloy.

It is generally accepted that hot tearing happens in the mushy zone with a lower volume fraction of eutectic phase at the final stage of solidification [35,36,42]. The lower the eutectic fraction, the more difficult it is to obtain a continuous feeding channel that is composed of a dendritic bridge and thick liquid film during the solidification process [7,9,27]. The good feeding channel contributes to the back-filling and healing of cracks, thus reducing the HTS of the alloy. Obvious tearing edges are observed on the fracture surface of alloys after Ca addition in Figure 5. Continuity of tearing edge corresponds to the change in eutectic content and liquid film thickness. The highest eutectic content and thicker liquid film generate the best uninterrupted tearing edge in AZ91–0.1Ca alloy (Figure 5c). Further research of fracture surface of the alloy was carried out by energy spectrum analysis, as shown in Figure 10. Ca is uniformly soluble in α-Mg matrix and Mg_17_Al_12_ phase, which can enhance the high temperature properties of dendrites and inter-dendrites. Moreover, many spikes considered as evidence of solid bridging between dendrites [20,46] are observed at the front of the tearing edge. This is a consequence of the propagation behavior of cracks along the liquid film between dendrites at the final stage of solidification. The liquid film is stretched under the tensile stress of volumetric shrinkage and torn apart at its forefront, forming dendritic separation morphology, ultimately with tearing edges and spikes. Therefore, the better the continuity of tearing edges and the higher the number of spikes is, the stronger is the ability to feed and deform the microstructure to resist hot tearing. The interdendritic bridging and solid-solution strengthening of Ca work together to enhance the bonding force between dendrites, which resulted in the lowest HTS in AZ91–0.1Ca alloy.

**Figure 11:**
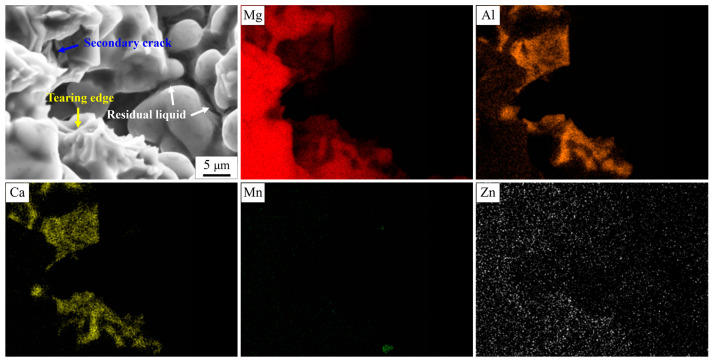
Energy spectrum analysis of the fracture surface of AZ91–1.0Ca alloy.

When the Ca addition is below 0.5 wt.%, these alloys exhibit excellent resistance to hot tearing. The added Ca, mainly in the form of substitutive solid solution, not only increases the eutectic content and its corresponding liquid film thickness, but also improves the strength of dendrites at high temperature [31,32,47]. Interdendritic bridging phenomenon (spikes) is clearly observed on the fracture surface, especially in the AZ91–0.1Ca alloy. However, the formation of Al_2_Ca phase decreases the eutectic content and its corresponding liquid film thickness with the increase in Ca content. The occurrence of a small amount of Al_2_Ca phases in AZ91–0.5Ca can still slightly improve the hot tearing resistance of AZ91 alloy, while a large amount of Al_2_Ca phases in AZ91–1.0Ca seriously deteriorate the hot tearing resistance of AZ91 alloy. It is deduced that the highest HTS of AZ91–1.0Ca alloy is closely related to the amount of Al_2_Ca phase. To further confirm the speculation, the amplified fracture surface and the corresponding energy spectrum of AZ91–1.0Ca was analyzed, as shown in Figure 11. The distribution of Ca in AZ91–1.0Ca is quite different from that in AZ91–0.1Ca alloy. It mainly concentrates near Al to form bulk Al_2_Ca. Meanwhile, only a few isolated tearing edges are observed on the surface of Al_2_Ca, suggesting that the residual melt is split into multiple independent small melt pools by the Al_2_Ca at the final stage of solidification. Owing to the high melting point and hardness of Al_2_Ca, its distribution in dendrites of boundaries will block the feeding channel of the eutecticum. Moreover, the significant difference in deformation capacity between Mg_17_Al_12_ and Al_2_Ca is prone to stress concentration at their interfaces during solidification shrinkage of the alloy [23,48]. Subsequent cracks nucleate and propagate along their interfaces with limited feeding melt. The results of Figure 5f and its EDS also demonstrate that the microcrack originated from the region of enriched Al_2_Ca phase. This is why AZ91–1.0Ca alloy has the worst hot tearing resistance, although its grain size is slightly smaller than that of AZ91.

**Figure 12:**
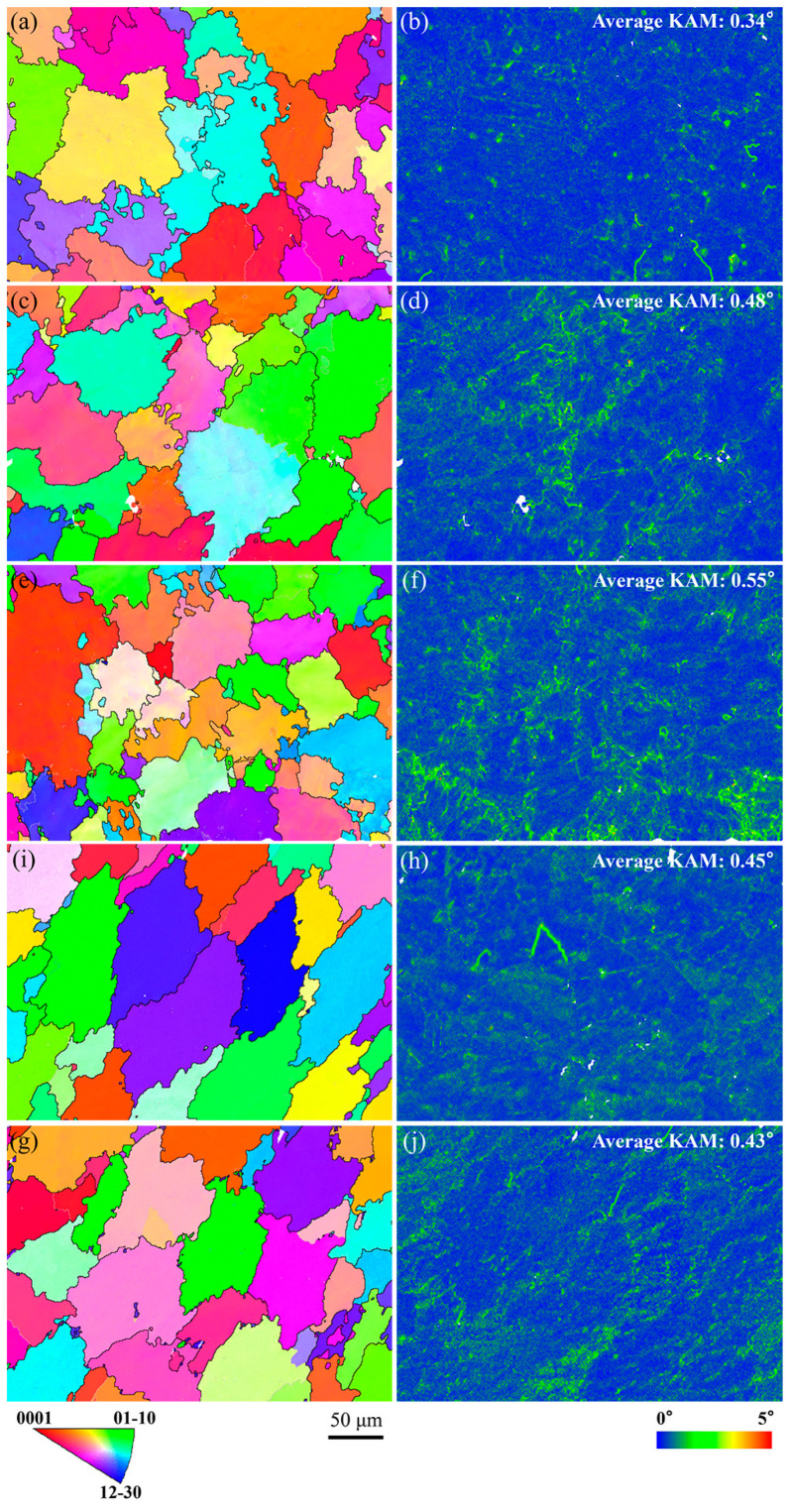
EBSD results of IPF map (**a**,**c**,**e**,**g**,**i**) and KAM map (**b**,**d**,**f**,**h**,**j**) for AZ91 (**a**,**b**); AZ91–0.05Ca (**c**,**d**); AZ91–0.1Ca (**e**,**f**); AZ91–0.5Ca (**g**,**h**); AZ91–1.0Ca (**i**,**j**).

Microscopic strain of the microstructure near the fracture surface for AZ91 alloy with Ca addition was evaluated by the average spatial distribution of KAM values, as shown in Figure 12. The average KAM of the microstructure is comparatively small for studied alloys, with a value below 0.6°. This happens because the as-casted alloy is only subject to the stress of volumetric shrinkage and die blocking during solidification. The average KAM increases first and then decreases slightly with the increase in Ca content in AZ91 alloy, and reaches its maximum in AZ91-0.1Ca alloy. This means that the solid-solution behavior of Ca improves the deformation ability of the microstructure. Clearly, the KAM mainly appears at grain boundaries due to the accumulation of shrinkage strain of the expelled solute during solidification. The solid-solution behavior Ca also strengthens the vulnerable areas between grains, resulting in a good intergranular bonding state. Hence, the largest KAM of AZ91–0.1Ca indicates the best resistance to deformation and hot tearing. The appearance of Al_2_Ca phase results in higher KAM of both AZ91–0.5Ca and AZ91–1.0Ca compared to AZ91. However, the aggregation distribution of Al_2_Ca phase between grains decreases the intergranular bonding force and promotes the nucleation of crack under stress concentration. This behavior is related to the secondary cracks found near the fracture, shown in Figure 4. Moreover, the grains are approximately equiaxed and without obvious orientation in the inverse pole figure (IPF) map. The tendency of grain size is consistent with the results in Figure 3 in the case of a relatively small statistical quantity of grains.

## 5. Conclusions

The HTS of AZ91 alloy with Ca addition of 0.05–1.0 wt.% was performed by a constraint rod casting method. Factors influencing hot tearing, such as grain size, eutectic content and corresponding liquid film thickness, were also investigated in detail to illustrate the hot tearing behavior of the alloy. The main results of this study are summarized as follows:

(1) With the increase in Ca content, the grain size was positively correlated with HTS of alloy and presented a ν-shaped tendency, while the eutectic volume fraction and liquid film thickness were negatively correlated with HTS and presented a ∧-shaped tendency. Compared with AZ91, a 37.5% decrease and 100% increase in HTS occurred with Ca addition of 0.1 wt.% and 1.0 wt.%, respectively.
(2) Alloys with Ca content not exceeding 0.1 wt.% exhibited excellent resistance to hot tearing. The solid-solution behavior of Ca in α-Mg matrix and Mg_17_Al_12_ phase increased the eutectic content and its corresponding liquid film thickness. A 16.3% increase and a 21.8% decrease in eutectic content appeared with Ca additions of 0.1 wt.% and 1.0 wt.%, respectively. It also strengthened the microstructure, especially the vulnerable areas between grains with higher KAM. The best resistance to deformation and hot tearing appeared in AZ91–0.1Ca alloy with the largest KAM. With further increases in Ca content, the formed Al_2_Ca phases underwent aggregation and coarsening, while the eutectic content and its corresponding liquid film thickness decreased. The coarsened Al_2_Ca at dendrite boundaries blocked the feeding channel and caused stress concentration, and eventually led to the nucleation of cracks.
(3) The hot tearing fracture surface consisted of a free dendritic plane, solute-rich secondary phase, tearing edges, spikes and microcracks. The occurrence of continuous tearing edges and many spikes in AZ91–0.1Ca alloy is attributed to the highest eutectic content and thicker liquid film. The fracture surface of AZ91–1.0Ca alloy only had a few isolated tearing edges separated by Al_2_Ca phase.
(4) The trace addition of Ca effectively improved the hot tearing performance of the alloy. This provides a new method for inhibiting hot tearing based on solid-solution strengthening and toughening. Due to the good solid-solution strengthening of most elements in magnesium alloys, this provides the possibility for the preparation of more hot tearing resistant alloys. Moreover, the addition of trace elements can achieve low-cost materials, which is crucial for cost reduction in the manufacturing industry.

## Figures and Tables

**Table 1:** Chemical composition of the AZ91 alloys with Ca addition (wt.%).

Alloy	Elements				
	Al	Zn	Mn	Ca	Mg
AZ91	8.94	0.67	0.20	–	Bal.
AZ91–0.05Ca	8.93	0.71	0.15	0.036	Bal.
AZ91–0.1Ca	9.41	0.70	0.21	0.089	Bal.
AZ91–0.5Ca	8.72	0.68	0.17	0.388	Bal.
AZ91–1.0Ca	9.29	0.85	0.14	0.904	Bal.

**Table 2:** Results of EDS analysis of AZ91–0.5Ca alloy in Figure 5f (at.%).

Point	Elements				
	Mg	Al	Zn	Mn	Ca
A	68.87	24.80	–	6.33	–
B	71.81	25.00	2.07	–	1.12
C	80.96	5.79	7.53	–	5.72

**Table 3:** EDS results of the secondary phase of AZ91–1.0Ca alloy in Figure 5f (at.%).

Point	Elements				
	Mg	Al	Zn	Mn	Ca
D	59.81	37.97	2.22	–	–
E	27.70	55.47	–	–	16.83
F	44.15	45.64	–	10.21	
G	67.77	30.75	1.48	–	–

**Table 4:** Mole fraction of solid (MFS) and temperature (T) corresponding to the turning point of solidification curves in Figure 8a.

Alloy	Point H		Point I		Point J		Solidification Interval (°C)
	MFS (%)	T (°C)	MFS (%)	T (°C)	MFS (%)	T (°C)	
AZ91	0.002	601.8	–	–	0.844	431.6	170.2
AZ91–0.05Ca	0.002	601.8	–	–	0.844	432.2	169.6
AZ91–0.1Ca	0.002	600.9	–	–	0.843	432.7	168.2
AZ91–0.5Ca	0.002	599.6	0.685	527.4	0.856	436.5	163.1
AZ91–1.0Ca	0.002	598.0	0.594	546.7	0.855	442.8	155.2

## Data Availability

The raw/processed data required to reproduce these findings cannot be shared at this time as the data also form part of an ongoing study.
